# The atypical cannabinoid O-1602 shows antitumorigenic effects in colon cancer cells and reduces tumor growth in a colitis-associated colon cancer model

**DOI:** 10.1186/2050-6511-13-S1-A23

**Published:** 2012-09-17

**Authors:** Julia Kargl, Johannes Haybäck, Angela A Stančić, Gunther Marsche, Ákos Heinemann, Rudolf Schicho

**Affiliations:** 1 Institute of Experimental and Clinical Pharmacology, Medical University of Graz, 8010 Graz, Austria; 2 Institute of Pathology, Medical University of Graz, 8036 Graz, Austria

## Background

Cannabinoids and the endocannabinoid system play an important role the protection against inflammation and cancer. O-1602, a synthetic cannabinoid with antiinflammatory properties, has little affinity to classical cannabinoid receptors but shows cannabinoid-like effects. In the present study, we were interested whether O-1602 produces antitumorigenic effects in colon cancer cells and whether it could reduce tumorigenesis in the colon *in vivo*.

## Methods

We used the cell lines HT-29 and SW480 to study the effect of O-1602 on viability and apoptosis in cancer cells. A mouse model of colitis-associated colon cancer was employed to study the effect of O-1602 on tumor growth *in vivo*.

## Results

Viability of HT-29 and SW480 cells was decreased and apoptosis was promoted by O-1602 in a concentration-dependent manner (0.1–10 µM). In the mouse model, treatment with O-1602 (3 mg/kg, i.p., 12x, every second day during a period of 3 weeks) reduced tumor area by 50% and tumor incidence by 30%. Histological scoring showed a significant decrease in tumor load. In tumor tissue, O-1602 decreased levels of phosphorylated activator of transcription factor 3 (pSTAT3) by 50% and tumor necrosis factor alpha (TNF-α) by around 45%. Treatment with O-1602 led to a ten-fold increase in the expression of the tumor suppressor p53.

## Conclusions

O-1602 exerts antitumorigenic effects by targeting colon cancer cells as well as proinflammatory pathways known to promote colitis-associated tumorigenesis, thus providing a novel insight into antitumorigenic mechanisms of atypical cannabinoids. As O-1602 is free of central sedation, it could be an interesting compound for the treatment of colon and possibly other cancers.

